# Jun‐Mediated Changes in Cell Adhesion Contribute to Mouse Embryonic Stem Cell Exit from Ground State Pluripotency

**DOI:** 10.1002/stem.2294

**Published:** 2016-02-13

**Authors:** Giulia Veluscek, Yaoyong Li, Shen‐Hsi Yang, Andrew D. Sharrocks

**Affiliations:** ^1^ Faculty of Life SciencesUniversity of ManchesterManchesterUnited Kingdom

**Keywords:** Embryonic stem cells, Jun, Adhesion, Fibronectin

## Abstract

Embryonic stem cells (ESC) are able to give rise to any somatic cell type. A lot is known about how ESC pluripotency is maintained, but comparatively less is known about how differentiation is promoted. Cell fate decisions are regulated by interactions between signaling and transcriptional networks. Recent studies have shown that the overexpression or downregulation of the transcription factor Jun can affect the ESC fate. Here we have focussed on the role of the Jun in the exit of mouse ESCs from ground state pluripotency and the onset of early differentiation. Transcriptomic analysis of differentiating ESCs reveals that Jun is required to upregulate a programme of genes associated with cell adhesion as ESCs exit the pluripotent ground state. Several of these Jun‐regulated genes are shown to be required for efficient adhesion. Importantly this adhesion is required for the timely regulated exit of ESCs from ground state pluripotency and the onset of early differentiation events. Stem Cells *2016;34:1213–1224*

**Significance Statement**

We are beginning to understand the gene regulatory mechanisms through which embryonic stem cells are able to remain in a pluripotent state. However, comparatively less is known about how embryonic stem cells exit from this state and begin to acquire new cellular identities. Here we have studied how the transcription factor Jun controls this process and uncover an important role for this protein in changing the adhesive properties of cells. This change in how cells interact with their external environment is important in promoting the loss of the embryonic stem cell state. This knowledge therefore enhances our understanding of how embryonic stem cells can be manipulated for potential therapeutic applications.

## Introduction

Mouse embryonic stem cells (ESCs) are an excellent model to study pluripotency and differentiation, and the findings can potentially be applied to human ESCs since they share many common features, such as the core pluripotency network (reviewed in 1). However, human ESCs share even more features with mouse epiblast stem cells (EpiSCs), including culture conditions and epigenetics 1. Mouse EpiSCs are cells isolated from the epiblast of the postimplantation blastocyst and are a more developmentally advanced stage than mouse ESCs (mESCs) 2, 3. mESCs can be maintained in a pluripotent ground state by using two pharmacological inhibitors (known as “2i”) to shield them from differentiation‐inducing stimuli transmitted from the Erk mitogen‐activated protein kinase pathway (MAPK) and by increasing the biosynthetic activity through inhibition of glycogen synthase kinase 3 (Gsk3) 4. More recently, an equivalent human ESC cell pluripotent ground state has been generated using a similar pharmacological inhibition strategy 5, and this ground state can be stabilized using further pharmacological inhibition, including JNK pathway inhibitors 6. JNK pathway inhibitors also stabilize ground stage pluripotency in monkey‐derived cells 7.

The undifferentiated state of ESCs is controlled at the transcriptional level and is characterized by the unique expression of a core of transcription factors, which are Oct4, Sox2, and Nanog 8. These master regulators alongside with other transcription regulators, such as Rex1, Myc, Klf4/5, and Tbx3, are the hubs of a complex network that regulates embryonic stem cell fate 8, 9, 10, 11, 12. Importantly, cell fate decision processes also require the orchestrated activation and repression of gene expression programs, involving loss of these transcription factor hubs and the production of new regulatory circuits 8. However, to date the mechanisms through which the MAPK and Gsk3 pathways regulate cell fate decisions are largely unknown. It is clear that the underlying chromatin environment shows large changes following exit from ground state pluripotency and the onset of cellular differentiation 13, 14 and transcription factors such as Otx2, Tcf3, Tcf15, and Tfe3 have recently been implicated in controlling this process 15, 16, 17, 18, 19. Recently, two RNA interference (RNAi) screens have uncovered the network of genes required for exit from ground state pluripotency, and transcriptional and chromatin regulators featured prominently among these genes 16, 20. One such gene encodes the transcription factor Jun 20.

Jun is a basic leucine zipper transcription factor member of the activator protein‐1 (AP‐1) family 21. The AP‐1 complex is involved in the transcriptional regulation of many cellular processes such as proliferation, inflammation, apoptosis, adhesion, migration, wound healing, cancer development, and metastasis 21, 22. Jun is phosphorylated by both Erk and Gsk3 kinases, which control its transactivation activity and stability 23, 24 and is suggestive of a link between Jun and these pathways during ESC differentiation. Moreover, Jun is also phosphorylated by Jnks, inhibition of which stabilizes ESC ground state pluripotency 6, 7, and the Jnk pathway plays an important role during stem cell differentiation 25, 26. Jun has been shown to be important in mouse development as mice without Jun cannot develop properly due to altered hepatogenesis, and die at 12–16 days postcoitum 27. Jun has also been linked to bone and cartilage formation 28, 29, myogenesis and vascular system development (reviewed in 22). mESCs lacking Jun exhibit a defect in differentiation into hepatocytes 27, and more recently a gain‐of‐function screen demonstrated that overexpression of Jun drives exit from pluripotency 30. This was recently confirmed and a role for Jun in blocking induced pluripotent stem cells (iPS) cell formation was demonstrated, further implicating Jun in a potential role in controlling the exit from the ESC state 31.

Although unbiased gain and loss of function approaches have converged on Jun as an important regulator of exit of ESCs from ground state pluripotency 20, 30, the mechanism of action of Jun in this context is not known. Here we have probed the functional role of Jun in ESCs and demonstrate that one major route through which Jun functions is through controlling the expression of a programme of genes associated with cell adhesion. Several of these genes are shown to be required for efficient adhesion and this adhesion is required for the timely and efficient exit of ESCs from ground state pluripotency.

## Materials and Methods

### Cell Culture and Adhesion Assay

E14 mESCs were routinely passaged as described previously 20. Cells were kept pluripotent by growing in the presence of the GSK3 inhibitor CHIR99021 and MEK inhibitor PD0325901 (“+2i” media) and allowed to exit from ground state pluripotency by changing the media to NDiff N2B27 lacking these pharmacological inhibitors.

EpiSCs were routinely passaged in a feeder‐free condition as described previously (32; kindly provided by Sally Lowell) with minor modifications. Briefly, EpiSC were grown in the N2B27 medium supplemented with 20 ng/ml Activin A (Peprotech 120‐14E, Rocky Hill, NJ, http://www.peprotech.com) and 10 ng/ml fibroblast growth factor 2 (R&D Systems 233‐FB‐02, Minneapolis, http://www.rndsystems.com) on plates coated with 7.5 μg/ml of human fibronectin (Sigma F1141, https://www.sigmaaldrich.com Saint Louis, Missouri).

For the adhesion assay, cells were detached nonenzymatically with 5 mM EDTA for 5 minutes, and equal numbers of viable cells replated and left to adhere for 3 hours before washing and counting adherent cells. Assessment of cell viability was performed using Trypan blue reaction and viable cells counted.

### RNA Extraction, RNA‐seq, RNA Interference, and Reverse Transcription Quantitative Polymerase Chain Reaction

RNA was extracted using the RNeasy Plus kit (Qiagen, Hilden, Germany, http://www1.qiagen.com) according to the manufacturer's instructions. For RNA‐seq, RNA was purified with one additional step of DNAse I treatment according to the manufacturer's instructions (QIAgen RNeasy kit). cDNA libraries from two biological replicates were prepared following the TruSeq Stranded mRNA sample preparation protocol (Illumina, https://www.illumina.com) and sequenced with an Illumina HiSeq. Data are available at ArrayExpress (E‐MTAB‐4068).

RNAi was performed using transiently transfected small interfering RNA (siRNA) as described previously 20 using ON‐TARGET plus siRNAs (Thermo Scientific, http://www.thermoscientific.com): Nontargeting Control (NTC) (D‐001810‐10‐05), *Jun* (L‐043776‐00‐0005), *Itgb1* (L‐040783‐01‐0005), *Itgav* (L‐046779‐01‐0005), and *Fn1* (L‐043446‐01‐0005).

Primers for reverse transcription quantitative polymerase chain reaction (RT‐qPCR) were designed using the PerlPrimer tool 33 and are listed in the Supporting Information Table S8. RT‐qPCR and multiplex RT‐qPCR were performed using the Fluidigm (https://www.fluidigm.com) BioMark HD System as described previously 19.

### Computational Analysis

For the RNA‐seq, reads (50 bp) were mapped to the mm9 mouse assembly genome using TopHat2 with the default settings and following the general workflow as described previously 34, 35. The mapped reads were then put through Cufflinks to assemble the transcripts and for statistical analysis of the differentially expressed genes, with a *p* value cut‐off of *p* < .01. Gene ontology analysis of the differentially expressed genes was performed using the DAVID Gene Functional Classification Tool 36. The intersect of outputs from MEME 37 and Homer 38 was used for high confidence AP1 motif discovery.

To compare gene expression changes occurring following Jun depletion and overexpression, we downloaded the data for gene expression levels (TPM) from mESCs treated with doxycycline for 36 hours to induce Jun (GSM1229024) and the corresponding control sample (GSM1229025) 31. We then obtained the intersection of the 460 significantly differentially expressed genes caused by Jun depletion (siJun) for 2 days, and the 20,325 expressed genes present in these datasets. This left a total of 406 genes and we calculated the fold changes between the Jun overexpressed sample and the corresponding control sample (adding 0.1 to all of the data to avoid infinite values for genes showing no expression under one condition). To prepare heatmaps, as the fold changes are in log_2_ scale, in order to make a fair comparison of the fold changes of siJun samples and c‐Jun overexpressed samples we did linear transformations separately to the positive and negative values of the c‐Jun overexpressed samples. This enabled the colour ranges of the positive and negative values of the fold changes obtained in Jun overexpression samples to be the same as those in the siJun‐depleted samples.

### Chromatin Immunoprecipitation

Chromatin immunoprecipitation (ChIP) was performed according to 39 using 1 μg of Jun antibody (Thermo‐Scientific PA1‐833) and 2 × 10^6^ cells. The IPed DNA was quantified with qPCR as described previously 40. Primers for ChIP‐PCR were designed using the online tool Primer3 41. For a negative control region; NC_F (ADS2765) AGGCTGGAAGGGTAGGTTGT and NC_R (ADS2764) GTGAATGTTGGCCTGATGTG, and for Fn1; ChIP_Fn1_F (ADS4842) CCATCGACTCTTGGGCTTTG and ChIP_Fn1_R (ADS4843) GGAACTTTAGGTCCTGGGCT.

### Western Blotting

Western blotting was performed with the following primary antibodies; Erk (Cell Signalling 4695, Beverly, MA, http://www.cellsignal.com), Jun (Thermo‐Scientific PA1‐833), Itgav (BD Bioscience, San Diego, http://www.bdbiosciences.com; 550024), Itgb7 (Santa Cruz, Santa Cruz, CA, http://www.scbt.com; 15330), and fibronectin (Sigma F3648). Secondary antibodies were IRDye 680LT anti‐Rabbit IgG and IRDye 680LT anti‐Rat IgG (Li‐Cor Biosciences). Signal was detected and quantified using the Odyssey Imaging System (Licor Biosciences). All Western blots are representative of at least three biological replicates.

## Results

### Jun Contributes to a Timely Regulated Exit from the Pluripotent State

**Figure 1:**
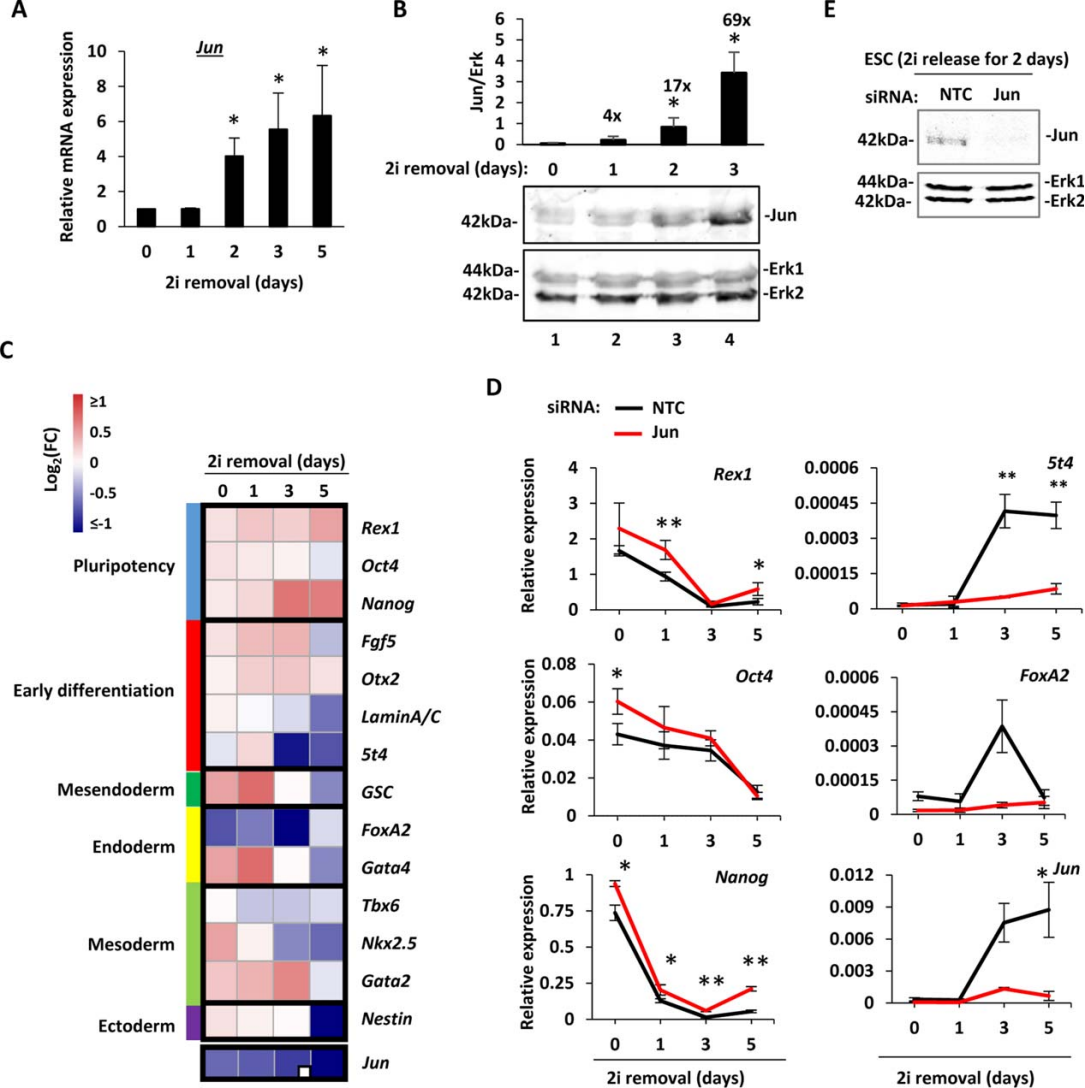
Jun expression and contribution to ESC state. **(A)**: Quantification of *Jun* mRNA levels after release from inhibition with 2i over a 5 day period. The values are normalized against *Gapdh* and are relative to day 0 (taken as 1). Error bars represent SEM (*n* = 3) (*, *p* < .05). **(B)**: Western blot analysis of Jun protein expression in ESCs upon release from culturing in 2i. Samples were collected at day 0 (lane 1‐in 2i), days 1, 2, and 3 (lanes 2–4). The upper panel shows Jun protein expression, and the bottom panel was blotted with anti‐Erk antibody as a loading control. Jun protein expression was quantified and normalized against Erk levels and is shown above the blots. The error bars represent the SEM (*n* = 3) (*, *p* < .05). The enrichment values compared to day 0 are indicated above the corresponding time points. **(C)**: ESCs were treated by transient transfection with either NTC or *Jun* siRNA, released from the 2i inhibition and the expression of pluripotency and differentiation‐associated genes was assessed at 0, 1, 3, and 5 days using the BioMark HD System (Fluidigm). The FC of the expression of each gene in the knockdown condition compared to the NTC condition are reported as a heatmap as log_2_(siRNA/NTC). For better visualization cut‐offs of log_2_ = 1 and −1 were chosen. Red values represent increases and blue represents decreases in gene expression following *Jun* knockdown. The marker genes have been grouped together depending on their known role, which is indicated as different colors on the left side of the heatmap. The sample number is *n* = 3 (or *n* = 2 when indicated with a white square on the bottom‐right corner). **(D)**: Examples of the expression profiles. Mean expression values are expressed as relative to the geometric mean of six housekeeping genes (*Gapdh, Ppia, Pgk1, Tbp, Tubb5*, and *Ywhaz*), and error bars represent SEM (*, *p* < .05; **, *p* < .01). Black lines represent the NTC siRNA treated samples, red lines represent the *Jun* siRNA treated samples. **(E)**: Western blot analysis of Jun expression in mESCs at day 2 after 2i release. The effect of Jun removal compared to the NTC siRNA is shown. Erk was used as loading control. Abbreviations: ESC, embryonic stem cell; FC, fold change; NTC, nontargeting control; siRNA, small interfering RNA.

To explore how Jun might affect ESC fate we first explored its expression as cells exit from ground state pluripotency. mESCs were maintained in the ground state by culturing with two pharmacological inhibitors CHIR99021 (for GSK3) and PD0325901 (for MEK) (2i) 4 and allowed to exit from the ground state by withdrawal of these inhibitors. *Jun* mRNA is expressed at low levels pluripotent ESCs cultured in 2i (Fig. 1A), and Jun protein is barely detectable by Western blot analysis of mESCs under these conditions (Fig. 1B). RT‐qPCR analysis of *Jun* transcript levels shows that *Jun* mRNA expression is strongly induced 2 days after 2i removal, and the mRNA level continues to increase at days 3 and 5 after 2i withdrawal (Fig. 1A). The increase in *Jun* mRNA expression reflects an increase in protein expression as Jun protein expression is increased in a statistically significant manner 2 days after release from 2i and increases further afterward (Fig. 1B).

Next we analyzed the mRNA expression of marker genes to understand the contribution of Jun to pluripotency and differentiation. *Rex1, Oct4*, and *Nanog* were used as markers of the pluripotent state 42, 43, 44, *Fgf5* and *Otx2* are upregulated as ESCs exit from the pluripotent state 19, 45 while other markers have a differential expression depending on the fate to which the cells commit (e.g., *Nestin* for ectoderm, *Tbx6* for mesoderm, *FoxA2* for endoderm) 46, 47, 48. To understand how Jun might contribute to the exit from pluripotency, the behavior of these marker genes upon *Jun* knockdown was assessed in a time‐course experiment carried out for 5 days after 2i withdrawal. In wild type cells *Jun* mRNA is greatly induced between 1 and 3 days after 2i removal but upon siRNA treatment *Jun* is no longer induced and remains at low levels during the 5 days (Fig. 1C, 1D). siRNA depletion also leads to loss of Jun protein (Fig. 1E). Upon *Jun* depletion, a small but statistically significant increase in the expression of pluripotency‐associated markers in ESCs in the pluripotent state (day 0) and a delay in their silencing upon 2i removal can be observed (Fig. 1C, 1D—*Rex1, Oct4* and *Nanog*). Conversely, following *Jun* depletion, many differentiation‐associated genes, such as *5t4* and *FoxA2*, show a delayed and/or a reduced induction especially at the later time points, during day 3 and day 5 (Fig. 1C, 1D; Supporting Information Table S1). These results are consistent with the observation that Jun is required for timely regulated exit from ground state pluripotency 20.

### Transcriptomic Analysis of the Genome‐Wide Role Played by Jun

**Figure 2:**
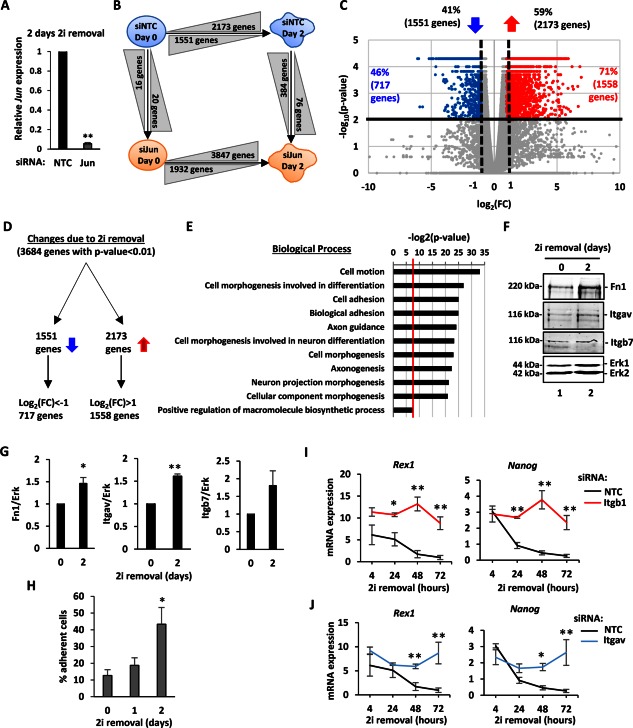
Upregulation of adhesion in differentiating embryonic stem cells (ESCs). **(A)**: Reverse transcripton quantitative polymerase chain reaction (RT‐qPCR) analysis of the knockdown efficiency of Jun by siRNA treatment. RNA expression 2 days after 2i removal was measured and normalized against *Gapdh* and is relative to NTC (*n* = 3) (**, *p* < 01). **(B)**: Schematic representation of the four conditions analyzed with an RNA‐seq experiment. Cells were treated with siRNA (either NTC, blue or against *Jun*, orange) and were released from the 2i inhibition for 2 days. RNA was collected either at day 0 (still in 2i) or at day 2 (*n* = 2). The arrows indicate the direction of the interaction studied. The number of genes that change between conditions are indicated in grey triangles, and triangles indicate the nature of the change: increasing height indicates upregulation, decreasing height indicates downregulation. A *p* value cut‐off of .01 was applied. **(C)**: The gene expression changes due to the 2i withdrawal are represented with a volcano plot. On the *x*‐axis the FC is reported as log_2_(FC), and on the *y*‐axis the *p* value is reported as −log_10_(*p* value). The horizontal line represents the *p* value threshold set at .01. The numbers and percentages of downregulated (left) and upregulated (right) are indicated above the graph. Vertical dashed lines represent the thresholds of log_2_(FC) = 1 or of log_2_(FC) = −1. The percentage and number of genes with *p* value < .01 and log_2_(FC) either > 1 or < −1 are indicated in the corresponding parts of the graph and individual data points colored red (for significantly upregulated) and blue (for significantly downregulated). **(D)**: Schematic representation of the partitioning of the gene expression changes. The directions of the blue arrows indicate upregulated and downregulated genes. **(E)**: Gene ontology term enrichment analysis for genes upregulated upon 2i removal. The biological processes are reported on the *y*‐axis, and the significance of the enrichment is reported on the *x*‐axis as log_2_(*p* value). A *p* value cut‐off of .01 was applied (red vertical line). **(F)**: Western blot analysis of Fn1, Itgav, and Itgb7 protein expression in ESCs in 2i (day 0 – lane 1) and 2 days after 2i release (day 2—lane 2). Erk was used as loading control. **(G)**: Quantification of the protein abundance from the Western blot analysis in (E). The expression values were normalized against Erk. Error bars represent the SEM (*n* = 3) (*, *p* < .05; **, *p* < .01). **(H)**: An adhesion assay was performed for ESCs cultured for the indicated times in the absence of 2i. The percentage of adherent cells is reported as cells attached compared to the NTC control. Error bars represent the SEM (*n* = 3) (*, *p* < .05). **(I, J)**: RT‐qPCR analysis of *Rex1* and *Nanog* expression upon *Itgb1* (H; red lines) or *Itgav* knockdown (I; blue lines) in ESCs cultured in the absence of 2i for the indicated times. Mean expression values are reported and compared to the NTC condition (black lines). The mRNA level is expressed as relative to the geometric mean of the housekeeping genes (*Gapdh, Ppia, Pgk1, Tbp*, and *Tubb5*), and error bars represent SEM (*n* = 3, *, *p* < .05; **, *p* < .01). Abbreviations: FC, fold change; Fn1, fibronectin 1; Itgav, Integrin alpha V; Itgb7, Integrin beta 7; NTC, nontargeting control.

To explore the role of Jun in the loss of ground state pluripotency, an RNA‐seq experiment was performed following Jun depletion from ESCs. Samples were taken from cells maintained in 2i (day 0) and 2 days after release from 2i in the presence and absence of siRNAs against Jun. Knockdown efficiency was confirmed at both the mRNA (Fig. 2A) and protein levels (data not shown). The gene expression changes across four conditions were compared, and the analysis indicates that among genes that show statistically significant changes in gene expression (*p* < .01): 3,724 genes are differentially expressed between day 0 and day 2 in cells treated with the NTC siRNA (2,173 genes are upregulated and 1,551 downregulated), 5,779 are differentially expressed between day 0 and day 2 upon Jun removal (3,847 genes are upregulated and 1,932 genes are downregulated), only 36 genes are differentially expressed between NTC and Jun depleted pluripotent cells (20 are upregulated upon Jun removal and 16 downregulated) and 460 genes are differentially expressed between NTC and Jun depleted cells at day 2 (76 genes are upregulated upon Jun depletion and 384 are downregulated) (Fig. 2B). Among the deregulated genes following Jun depletion many of the markers of pluripotency and differentiation used to study the overall contribution of Jun to ESC fate (Fig. 1C, 1D) show similar behavior in this RNAseq experiment (Supporting Information Tables S2, S3).

**Figure 3:**
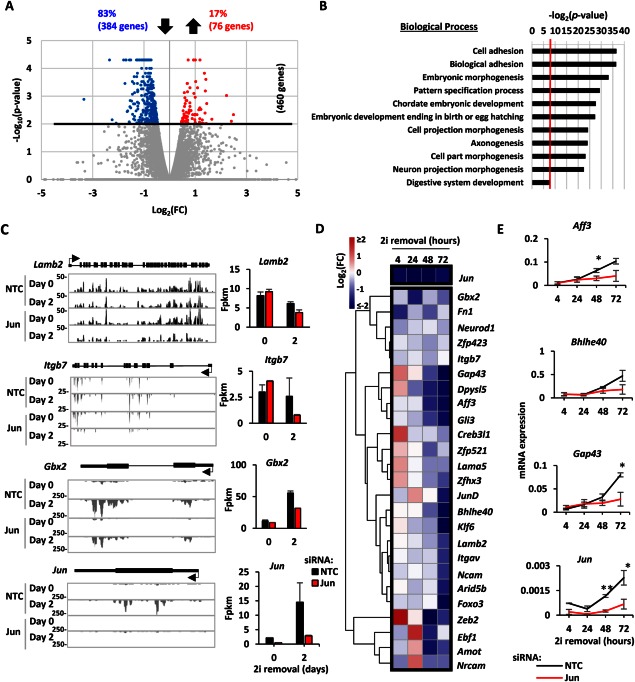
Contribution of Jun to embryonic stem cell (ESC) adhesion. **(A)**: Volcano plot representation of the gene expression changes due to *Jun* knockdown as described in Figure 2B. In this case all the genes scored log_2_(FC) > 0.5 so no vertical threshold was applied. **(B)**: Gene ontology Term analysis for the genes downregulated upon *Jun* removal. The biological processes are reported on the *y*‐axis, and the significance of the enrichment is reported on the *x*‐axis as log_2_(*p* value). A *p* value cut‐off of .01 was applied (red vertical line). **(C)**: UCSC Genome Browser tracks for the indicated genes are reported as examples of the effect of *Jun* removal. The height, the time point (day 0 or day 2), and the condition (NTC or *Jun* siRNA treated) for each track are reported on the left. Gene structure is shown in black above the tracks (thick line = exons, thin lines = UTR, the arrow indicates the transcription start site [TSS], and the direction of transcription). On the right side the fpkm values for each gene are reported. Error bars represent the SEM (*n* = 2). **(D)**: ESCs were treated with either NTC or Jun siRNA and released from the 2i inhibition. Gene expression was assessed at 4, 24, 48, 72 hours using the BioMark HD System (Fluidigm) as described in Figure 1C. **(E)**: Examples of the expression profiles for the experiment in (D) are shown as described in Figure 1D. Error bars represent the SEM (*n* = 3) (*, *p* < .05; **, *p* < .01). Abbreviations: FC, fold change; fpkm, fragments per kilobase per million sequenced reads; NTC, nontargeting control; siRNA, small interfering RNA.

To focus on genes that show larger changes in expression upon exit from the ground state, a second cut‐off was applied to identify those genes that change their expression by twofold or more (Fig. 2C, 2D; vertical dashed lines). Hence 71% of the upregulated genes (1,558 out of 2,173) and 41% of the downregulated genes (717 out of 1,551) were selected as genes whose expression changes upon 2i removal with a high statistical significance. Within the 717 genes downregulated upon 2i withdrawal there is an enrichment of factors involved in regulation of transcription and negative regulation of cell differentiation (Supporting Information Table S4). For instance, *Nanog, Esrrb, Sox2*, and *Klf4* are found among the significantly downregulated genes, and these have all been previously linked to pluripotency 9, 11, 49, 50 and confirms that the cells are exiting from ground state pluripotency. Within the 1,558 genes upregulated upon 2i withdrawal there is an enrichment of factors involved in cell motion and cell adhesion (Fig. 2E). Furthermore, there is an enrichment of factors involved in cell morphogenesis with particular propensity toward axonogenesis and neuronal development. The genes upregulated during differentiation are enriched in factors expressed on the cellular membrane, extracellular matrix (ECM), and cell‐cell junctions (Supporting Information Table S4). Therefore, as ESCs begin to lose their pluripotency as cells exit the pluripotent state, genes associated with cell motion and adhesion processes begin to become upregulated. Indeed, Western blot analysis of proteins known to be involved in cell adhesion (*Fn1, Itgav*, and *Itgb7*) confirms the upregulation of several of these genes at the protein level (Fig. 2F, 2G). Importantly, an adhesion assay performed on ESCs as they exit the ground state demonstrated a change of adhesive properties at the functional level (Fig. 2H). Inversely, correct expression of adhesion‐associated proteins is required for a timely exit from the pluripotent state. Itgb1 has been previously shown to be important for embryogenesis and ESC differentiation 51, and depletion of Itgb1 blocks the loss of Rex1 and Nanog expression following 2i removal (Fig. 2I). Similarly, we show that depletion of another integrin, Itgav (a putative Jun target gene; see Fig. 3D) blocks the loss of pluripotency gene expression which accompanies ESC exit from ground state pluripotency (Fig. 2J). Together, these results demonstrate that a major category of genes associated with cell adhesion are upregulated during the early phases of ESC exit from ground state pluripotency, and these genes play an important role in facilitating this exit.

### Jun‐Mediated Transcriptional Regulation Controls Adhesion in ESCs Exiting the Ground State

Jun has previously been linked to controlling cell adhesion and motility (reviewed in 21, 22). Therefore, to begin to analyze whether Jun has any effect on potentially controlling the changes in cell adhesion, we further analyzed our transcriptomic data. In agreement with Jun being an activator of transcription, a larger group of genes is downregulated than upregulated upon Jun removal: 76 genes are upregulated (17%) whereas 384 genes are downregulated (83%) with a *p* value < .01 (Figs. 2A, 3A). In this case the second cut‐off on log_2_(FC, fold change) is not applied since more than half of the genes with a *p* value < .01 show log_2_(FC) between −1 and −0.45 or between 0.45 and 1 (Supporting Information Table S3).

Gene ontology term analysis of the 384 genes downregulated upon Jun depletion shows an enrichment of factors involved in cell and biological adhesion, morphogenesis and embryonic development (Fig. 3B). Consistent with an association with adhesion, the genes downregulated upon Jun removal are enriched in factors expressed on the plasma membranes and cellular junctions (Supporting Information Table S5). However, we do not observe significant differences in the levels of the transcripts encoding E‐Cadherin and β‐Catenin, indicating that these important factors in driving cell‐cell contacts are therefore likely not important contributors to the Jun‐mediated changes in cell adhesion that we observe. The genes downregulated upon Jun removal have similar but not identical GO‐terms to the genes that are generally upregulated during differentiation suggesting that subsets of the biological processes are regulated by Jun. There are two different classes of transcription profiles of genes downregulated upon Jun removal (Fig. 3C). Some genes such as *Lamb2* and *Itgb7* are similarly expressed between day 0 and day 2, but their expression decreases at day 2 after Jun removal. Other genes such as *Gbx2* are highly expressed at day 2 compared to day 0 and their expression at day 2 decreases upon Jun removal.

Due to their high enrichment among Jun‐regulated genes, we further focussed on genes associated with adhesion. To validate the role of Jun in controlling this class of genes, we extended our analysis to additional time points (4, 24, 48, and 72 hours) and performed RT‐qPCR analysis on a panel of genes associated with the adhesion‐related GO terms (Supporting Information Table S6). The differences between the *Jun* knockdown and the NTC conditions are described in a heatmap format (Fig. 3D). Negative changes in gene expression tend be initiated upon Jun removal after 24 hours upon 2i removal and become more apparent after 2 and 3 days. Examples of expression profiles of genes affected by Jun removal include *Aff3, Bhlhe40*, and *Gap43* (Fig. 3E). In conclusion, validation studies confirmed that in differentiating ESCs Jun removal impairs the expression of adhesion‐associated genes with greater effects observed the longer cells were removed from 2i inhibition.

To provide further evidence for a role of Jun in ESCs as they exit from ground state pluripotency, we took advantage of a gene expression dataset derived from ESCs containing a doxycycline‐driven Jun transgene 31. In this study, ESCs were maintained in 2i and leukemia inhibitory factor (LIF) to maintain their pluripotent state, and Jun was subsequently overexpressed under these conditions. We therefore compared the expression of genes which changed upon depletion of Jun (Fig. 3A) with the changes in expression seen upon Jun overexpression. Importantly, the majority of genes that showed decreased expression upon Jun depletion showed reciprocal increases in expression upon Jun overexpression (266 from 358; Supporting Information Fig. S1A; Table S7). Conversely, the majority of genes that showed increased expression upon Jun depletion showed reciprocal decreases in expression upon Jun overexpression (36 from 48; Supporting Information Fig. S1A; Table S7). These distributions were significant (Supporting Information Fig. S1B) further validating our gene expression data and further supporting a role for Jun as a transcriptional activator in this context. Importantly, when considering the genes which consistently change across both experimental settings according to a role for Jun acting as a transcriptional activator, terms associated with “adhesion” are again found among the highest enriched GO categories (Supporting Information Fig. S1C).

**Figure 4:**
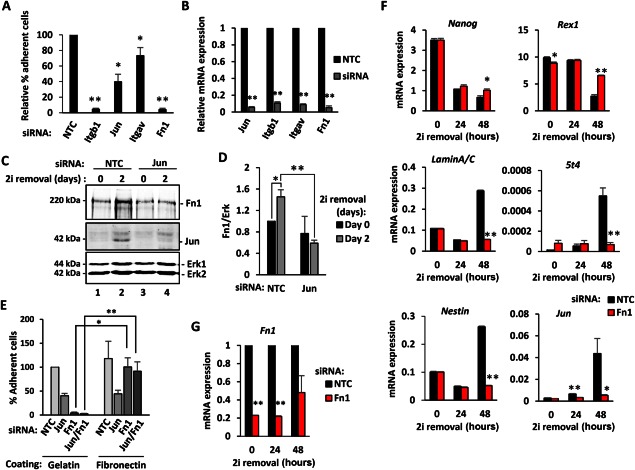
Jun regulates of embryonic stem cell (ESC) adhesion through Fn1. **(A)**: Adhesion of cells is reported as percentage of adherent cells relative to control cells (NTC siRNA) upon *Jun, Itgb1, Itgav*, and *Fn1* siRNA treatment. Error bars represent the SEM (*n* = 3) (*, *p* < .05; **, *p* < .01). **(B)**: Knockdown efficiency of the experiment in (A) is shown. RNA expression was normalized against *Gapdh* and is relative to NTC (*n* = 3) (**, *p* < .01). **(C)**: Western blot analysis of Fn1 expression in mouse ESCs at day 0 (lanes 1 and 3) or at day 2 (lanes 2 and 4) after 2i release. The effect of Jun removal (lanes 3 and 4) was compared to the NTC (lanes 1 and 2). Erk was used as loading control. **(D)**: Quantification of Fn1 from the Western blot analysis described in (C). Fn1 expression was normalized against Erk levels and it is relative to NTC at day 0 (taken as 1). Error bars represent the SEM (*n* = 3) (*, *p* < .05; **, *p* < .01). **(E)**: Adhesion of cells is reported as percentage of adherent cells relative to control cells (NTC siRNA) on gelatin coated plates, upon *Jun, Fn1* or *Jun* and *Fn1* siRNA treatment. Cells were grown on either gelatin or fibronectin coated plates. Error bars represent the SEM (*n* = 3) (*, *p* < .05; **, *p* < .01). **(F, G)**: Reverse transcription quantitative polymerase chain reaction analysis of pluripotency and differentiation markers, *Jun* (E) and *Fn1* (F) upon *Fn1* knockdown (red bars) are shown and compared to the NTC condition (black bars). The mRNA level is expressed as relative to the geometric mean of the housekeeping genes (*Gapdh, Ppia, Pgk1, Tbp*, and *Tubb5*), and error bars represent SEM (*n* = 3, *, *p* < .05; **, *p* < .01). Abbreviations: Fn1, fibronectin 1; Itgav, Integrin alpha V; Itgb7, Integrin beta 7; NTC, nontargeting control; siRNA, small interfering RNA.

To establish the functional requirements for Jun for appropriate adhesion of ESCs, we performed an adhesion assay upon depletion of Jun or several of its target genes (Fig. 4A, 4B). Without Jun only 40% of ESCs adhere compared to control NTC treated cells (Fig. 4A). Itgb1 was used as positive control, and only 10% of ESCs attach upon Itgb1 removal. We also tested Itgav, Gli3, and Fn1 in this assay, which are adhesion‐associated factors whose expression is downregulated upon *Jun* removal. Knockdown of all of these genes reduces cellular adhesion albeit to different degrees (Fig. 4A). Due to the strong effects on cell adhesion we next focussed further on *Fn1* which encodes fibronectin. Fn1 expression is significantly reduced upon Jun depletion at both the mRNA level (Fig. 3D) and at the protein level (Fig. 4C, 4D). However, while precoating the plates with fibronectin can rescue adhesion in cells treated with siRNAs against *Fn1*, precoating the plates with fibronectin is insufficient to rescue the adhesion defects seen upon loss of Jun expression, reflecting the broader role of Jun in regulating other adhesion associated factors (Fig. 4E). To establish whether fibronectin‐mediated adhesion has a role in controlling the exit of ESCs from ground state pluripotency, we monitored the progression of ESCs away from ground state pluripotency following depletion of *Fn1* (Fig. 4F, 4G). Upon *Fn1* removal, ESCs fail to upregulate differentiation markers such as *5t4, LaminA/C*, and *Nestin* and delay the downregulation of pluripotency markers such as *Rex1* and *Nanog*. Interestingly, cells that do not express *Fn1* cannot upregulate *Jun* expression upon 2i removal, suggesting that a positive feedback mechanism might exist between fibronectin and *Jun*.

Collectively, these data provide strong evidence to support a role for Jun‐mediated gene activation in controlling the expression of genes associated with cell adhesion and ultimately leading to changes in cell adhesion. These adhesive changes are important for exit from ground state pluripotency and the onset of cellular differentiation.

### Jun‐Mediated Gene Expression Changes Control the Adhesion of EpiSCs

**Figure 5:**
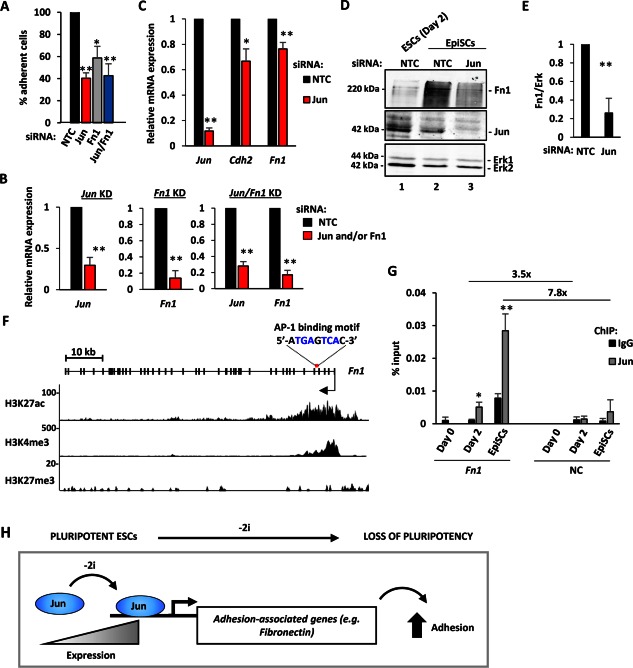
Contribution of Jun to adhesion of EpiSCs. **(A)**: Adhesion of EpiSCs was tested upon *Jun* and *Fn1* removal and is shown relative to NTC (taken as 100%). Error bars represent the SEM (*n* = 3) (*, *p* < .05; **, *p* < .01). **(B)**: KD efficiency of *Jun* and *Fn1* in the experiment described in (A). RNA expression was normalized against *Gapdh* and is relative to NTC (taken as 1) (*n* = 3) (**, *p* < .01). **(C)**: Expression of the indicated genes in EpiSCs upon *Jun* removal. The mRNA level was normalized against the geometric mean of the housekeeping genes (*Gapdh, Ppia, Pgk1, Tbp*, and *Tubb5*) and is relative to the control (NTC siRNA) condition (equal to 1; dashed line). Error bars represent the SEM (*n* = 3) (**, *p* < .01). **(D)**: Western blot analysis of Fn1 protein expression (top panel) in EpiSCs following NTC (lane 2) and Jun (lane 3) siRNA treatment. Cell lysate from ESCs at day 2 (NTC) was used as control (lane 1). Jun KD efficiency (middle panel) and Erk (loading control; bottom panel) are shown. **(E)**: Quantification of Fn1 levels from the Western blot analysis described in (B). Fn1 expression was normalized against Erk and it is relative to NTC (taken as 1). Error bars represent the SEM (*n* = 3) (**, *p* < .01). **(F)**: UCSC genome browser view of the *Fn1* gene structure (exons are represented with thick lines and introns with thin lines; the arrow indicates the TSS and the direction of transcription). The AP‐1 motif identified with HOMER and MEME, and its position is indicated above the *Fn1* gene. Primers for ChIP‐qPCR were designed around this motif. Histone modifications in ESCs (Yang et al. 19) are shown below. The type of histone modifications and the track height are indicated on the left. **(G)**: ChIP‐quantitative polymerase chain reaction analysis of Jun binding (light grey) to the *Fn1* locus in ESCs (day 0 and day 2 after 2i withdrawal) and EpiSCs. Pull down efficiency is expressed as % input and is compared to normal rabbit IgG control. Error bars represent the SD (*n* = 3) (*, *p* < .05; **, *p* < .01). The enrichment of Jun binding on *Fn1* relative to a NC region and is reported in the top part of the graph. **(H)**: Schematic representation of the findings. Jun levels increase as ESCs exit from ground state pluripotency, leading to the activation of a programme of genes associated with cell adhesion. This in turn contributes to the loss of pluripotency and the onset of the commitment and differentiation process. Abbreviations: AP‐1, activator protein‐1; ChIP, chromatin immunoprecipitation; EpiSC, epiblast stem cells; ESCs, embryonic stem cells; Fn1, fibronectin 1; KD, knockdown; NC, negative control; NTC, nontargeting control; siRNA, small interfering RNA.

Jun begins to be expressed between 1 and 2 days after release from 2i inhibition, and this time period corresponds to the time where cells start to become irreversibly committed to exit from ground state pluripotency. One of the first type of cells identified along the differentiation lineage, are the EpiSCs, which correspond to a more developmentally advanced stage 3, 52. We therefore asked whether Jun also regulates the adhesive properties of EpiSCs. *Jun* was depleted using siRNAs (Fig. 5B), and loss of *Jun* causes a 60% decrease in the numbers of adherent cells (Fig. 5A). Adhesion of EpiSCs is also reduced by depletion of the Jun target gene *Fn1* or by a combination of *Jun* and *Fn1* depletion (Fig. 5A). Thus both Jun and its target gene *Fn1* are involved in adhesion of EpiSCs. We therefore focussed further on fibronectin and asked whether its expression is regulated by Jun in EpiSCs. Jun depletion in EpiSCs caused reduced expression of *Fn1* and another adhesion associated gene *Cdh2* at the mRNA level (Fig. 5C). Cdh2, or N‐Cadherin, has been shown to regulate cell adhesion and to be important during gastrulation and embryonic left‐right asymmetry establishment 53, 54. Importantly, the reduction in *Fn1* expression was also mirrored at the protein level following *Jun* depletion (Fig. 5D, 5E). To provide a direct link between Jun and *Fn1* regulation we asked whether there were any Jun‐bound AP1 motifs present in the *Fn1* locus. Motif searching identified an AP‐1 motif within an intronic region located toward the 5′ end of the *Fn1* gene (Fig. 5F). This region is characterized by histone modifications which are associated with active promoter regions (Fig. 5F; 19). To establish whether Jun bound to this region in ESCs and EpiSCs, we performed ChIP and observed a progressive increase of Jun binding at the *Fn1* locus as cells transitioned from ground state pluripotency in 2i, through exit from the ground state for 2 days and reached the highest levels in EpiSCs (Fig. 5G). Importantly, Jun binding was specific as little signal was observed with nonspecific IgG control or to a negative control (NC) region. Thus Jun binds to the *Fn1* locus and this binding likely directly mediates the effects of Jun in controlling *Fn1* expression. Together these results demonstrate that Jun is required for adhesion in both ESCs as the exit from ground state pluripotency and EpiSCs and part of this activity is likely mediated through controlling *Fn1* expression.

## Discussion

The transcription factor Jun has a myriad of biological functions (reviewed in 21, 22) and was identified in a genome‐wide RNAi screen as a factor required for efficient exit of mESCs from ground state pluripotency and the onset of early differentiation events 20. Here we analyzed the transcriptomic changes that ESCs undergo following exit from ground state pluripotency and identified Jun‐mediated control of cellular adhesion as an important mechanism contributing to the early differentiation of ESCs.

Our transcriptomic analysis revealed the upregulation of different classes of genes contributing to multiple biological processes in mESCs upon exit from ground state pluripotency. Expected GO term categories were identified containing transcripts involved in cell morphogenesis and cell proliferation that were upregulated and downregulated, respectively. However, among the upregulated genes, there is also a considerable enrichment of processes involved in neural aspects of morphogenesis and differentiation. This is likely related to the culturing of the cells in N2B27 medium which is typically used for the neural differentiation of mESCs 55. More intriguingly, multiple GO term categories were highly enriched in processes related to cell adhesion, and this is consistent with the changes expected as ESCs begin to differentiate and go through an epithelial to mesenchymal transition. Moreover, cell‐cell adhesion through E‐cadherin has previously been shown to play an important role in maintaining pluripotency 56, 57. Genes contributing to cell adhesion also contributed to the most highly enriched GO term categories that were deregulated following Jun depletion. We also observe small increases in the expression of genes encoding pluripotency markers under 2i culture conditions which may reflect either a role for low level Jun expression in mESCs in their ground state or alternatively a reduction in spontaneous low level loss of pluripotency in these cultures. Importantly, we also show that Jun not only affects ESC state at early stages in the first few hours after 2i removal and early exit from the ground state 20 but also several days later after commitment to exit the ground state has passed. This is consistent with the observation that Jun expression gradually increases as ESCs exit the ground state and the protein becomes readily detectable between 24 and 48 hours after 2i withdrawal (Fig. 1) and is also seen by others in ESC differentiation systems 31. Importantly, although Jun controls the expression of factors that are involved in adhesion and motion, the exact GO terms and genes involved are not identical to those identified following exit of ESCs from ground state pluripotency in the presence of Jun. Therefore Jun is responsible for regulating a unique repertoire of genes associated with cell adhesion during this cell fate transition. Indeed, Jun depletion causes a decrease in the expression of proteins mainly found on the plasma membrane, in cell‐cell and cell‐substrate junctions and the ECM rather than intracellular signalling proteins suggesting a role more focussed on cell interactions with the ECM. For example, decreases in the genes encoding fibronectin (*Fn1*), collagens (*Col4a1* and *Col4a2*), laminins (*Lamb2, Lama5, Lamc1* and *Ntn1*), perlecan (*Hspg2*), versican (*Vcan*), and others extracellular matrix proteins (*Vwa1* and *Frem2*) was observed.

A role for Jun in controlling cell adhesion is consistent with the fact that Jun has recently been linked to controlling cell adhesion as Schwann cells are reprogrammed to repair cells during nerve injury 58. Jun is also required for wound healing and eyelid closure during mouse development, processes that require changes to adhesion and motility 59 Moreover, adhesion to the ECM has been shown to influence cellular differentiation in several ways, including studies in stem cells including ESCs (reviewed in 60). Here we now link these two findings in the context of a role for Jun‐mediated adhesion control in mESCs. Importantly, many of the genes identified in our study as regulated by Jun have been previously shown to cause embryonic lethality when not functional or removed from the developing embryos, including *Fn1, Lamb2, Lama5, Lamc2, Col4a1*/*a2, Hspg2, Vcan* 60. More generally, the integrin pathway and the ECM have been shown to be involved in many different biological processes, such as adhesion, spreading, proliferation, survival, morphogenesis, and differentiation of ES cells 61, 62. Here we demonstrate a role for Jun in maintaining the activation of genes encoding integrins (*Itga9, Itgav*, and *Itgb7*) and other intracellular members of the integrin signalling pathway (e.g., *Tln1*, Phospholipase C, and receptor tyrosine kinases such as *Ddr1, Flt1* and *Ptk7*).

Various proteins that compose the ECM are bound by integrins including fibronectin, laminins, and collagens 62. Here we show that fibronectin (Fn1) expression is controlled by Jun, and that reducing fibronectin expression prevents ESCs from exiting the ground state in a timely manner and upregulating markers of differentiation upon 2i removal. This suggests that fibronectin is not only important for the efficient adhesion of ESCs but also plays a role in fine tuning their state during differentiation. Fibronectin is a glycoprotein of the ECM that can bind both ECM proteins (such as collagen, fibrin and itself) as well as integrins 63. It is possible that integrin‐mediated signalling is responsible for the effect of fibronectin in promoting elevated Jun expression (either directly or indirectly) upon exit from ground state pluripotency. During embryogenesis, cell adhesion to fibronectin is required for proper mesoderm precursor maintenance and proliferation 64, and removal of fibronectin causes embryonic lethality 64. Furthermore, consistent with our loss of function studies, a previous investigation using different plating regimes identified that binding to fibronectin promotes ESC differentiation 61. Interestingly others have shown that mESCs generate their own fibronectin and this is important not only for differentiation (at high concentrations) but also for both promoting stem cell renewal (at intermediate concentrations) 65. Although fibronectin binding is clearly important in ESC cell fate choice, it is not yet known how fibronectin exerts this function in ESCs, although it is possible that the signalling is mediated by the binding to α5β1 and αv integrins, the principal fibronectin receptors 66. Indeed, such a hypothesis is supported by the observation that removal of either β1 or αv integrin affects the ability of ESC to exit from the pluripotent state, as demonstrated by their retention of the expression of pluripotency markers. This is consistent with previous data showing a role for αv integrin in human ESC function 67 and a role for αv integrin in maintaining the epistem cell‐like state in mESC cultures 68. Moreover, α5β1 integrin has a role in hESC function through adhesion to fibronectin 67, 69.

In addition to a role in ESCs, we also show that Jun removal affects adhesion‐associated gene expression in EpiSCs. Indeed, Jun is required for adhesion of EpiSCs and in both ESCs exiting from pluripotency and EpiSCs, Jun is required for the correct expression of fibronectin. These observations suggest that the role of Jun is at least partially conserved in the later stage of differentiation found in EpiSCs.

## Conclusion

In conclusion, the findings presented in this study suggest that rather than a role for Jun in differentiating ES cells by directly affecting the expression of the pluripotency network or differentiation‐promoting factors, it plays a major role in determining the correct induction and/or execution of other biological processes, such as adhesion and this function is required for efficient exit of ESCs from ground state pluripotency and the onset of early differentiation.

## Author Contributions

G.V.: conception and design, collection and/or assembly of data, data analysis and interpretation, manuscript writing, and final approval of manuscript; Y.L.: data analysis and interpretation, and final approval of manuscript; S‐H.Y. and A.D.S.: conception and design, data analysis and interpretation, manuscript writing, and final approval of manuscript.

## Disclosure of Potential Conflicts of Interest

The authors indicate no potential conflicts of interest.

## Supporting information

Additional Supporting Information may be found in the online version of this article

Supporting Information

Supporting Information
