# Supplementary material for: Jun‐Mediated Changes in Cell Adhesion Contribute to Mouse Embryonic Stem Cell Exit from Ground State Pluripotency
Source: Stem Cells. 2016 Feb 13;34(5):1213–24. doi: 10.1002/stem.2294 (PMC4864893; doi:10.1002/stem.2294)
Supplement: Supplementary file 1 — Supporting Information [file STEM-34-1213-s001.pdf]

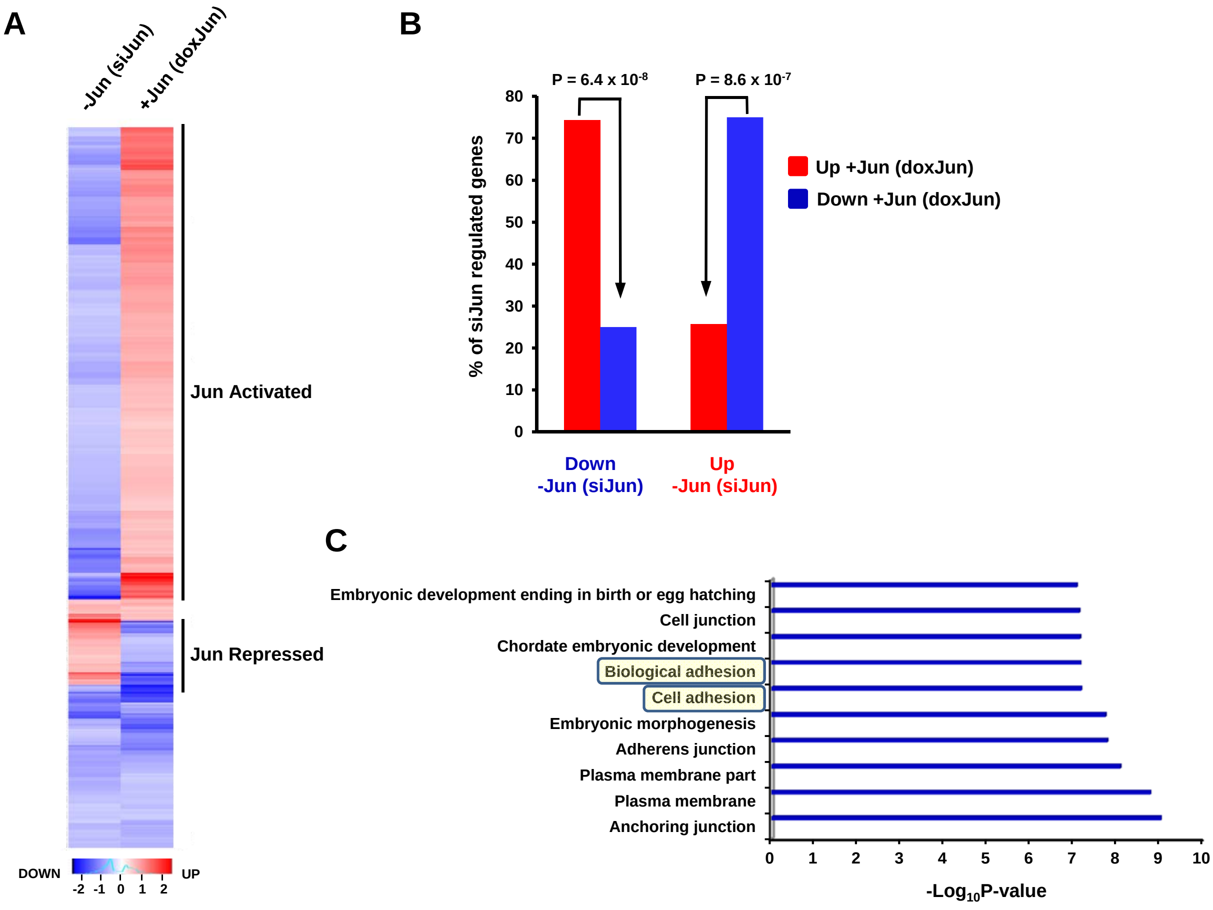

Supplementary Fig. S1: Reciprocal changes in gene expression are seen upon Jun depletion and overexpression. (A) Heatmap representation of the gene expression changes seen in ESCs 2 days after removal of 2i following siRNA-mediated Jun depletion (-Jun) (left column) and 36 hrs after doxycycline (dox)-mediated upregulation of Jun (+Jun) in ESCs maintained in 2i + LIF (right column). Groups of genes showing reciprocal changes in gene expression consistent with Jun acting as an activator or repressor are bracketed. **(B)** Summary of gene expression changes affected by Jun depletion (-Jun) and Jun over-expression (+Jun). P-values are calculated from a hypergeometric distribution. **(C)** Gene ontology analysis of the genes downregulated upon Jun removal and also upregulated upon Jun over-expression. The biological processes are reported on the y-axis and the significance of the enrichment is reported on the x-axis as -Log_{10}(P-value). The top ten GO terms are shown. Terms associated with “adhesion” are highlighted.

Supplementary Table legends

Further details are provided in the legends tab of the Excel spreadsheet.

Table S1. RT-PCR analysis of pluripotency and differentiation marker genes. Mean and standard error of the mean (SEM) values are shown for RT-qPCR data from the Fluidigm system (n=3) for the indicated genes under conditions of treatment with siRNAs against Jun (columns D and E) or a non-targeting control (NTC) (columns B and C). Samples are taken from mouse ESCs at 0, 1, 3 and 5 days (d) after 2i withdrawal. Expression levels differing significantly between the two conditions are coloured yellow (P-value <0.05) and red (P-value <0.01). Data are shown graphically on the right.

Table S2. Total expression values from RNAseq data in the presence and absence of Jun depletion. The expression of the indicated genes is shown (as RPKM values) for mouse ESCs grown in the presence of 2i (d0), or following 2i withdrawal for 2 days (d2) in the presence of non-targeting control (NTC) siRNA or siRNA against Jun. Values from each of the duplicate samples are shown.

Table S3. Relative expression values from RNAseq data in the presence and absence of Jun depletion. Pairwise comparisons of the RNAseq data in Supplementary Table S2 (samples being compared are indicated in each block of columns). Average expression values are shown in the first two columns of each block. Fold changes between the indicated samples and statistical significance are shown in the columns 3-5 in each block. Data are ranked according to the fold change initiated by Jun depletion 2 days after 2i withdrawal compared to NTC-treated samples.

Table S4. GO terms associated with genes whose expression changes following withdrawal of 2i. Significantly over-represented GO terms are shown for genesets that are upregulated (left) or downregulated (right) following 2i removal for 2 days.

Table S5. GO terms associated with genesets deregulated upon Jun depletion. Significantly over-represented GO terms are shown for genesets that are upregulated (left) or downregulated (right) following Jun depletion compared to NTC-treated cells, 2 days after 2i withdrawal.

Table S6. RT-PCR analysis of adhesion-associated genes. Mean and standard error of the mean (SEM) values are shown for RT-qPCR data from the Fluidigm system (n=3) for the

indicated genes under conditions of treatment with siRNAs against Jun (J; lines 5-8 in each block) or a non-targeting control (N; lines 1-4 in each block). Samples are taken from mouse ESCs at 4, 24, 48 and 72 hours (d) after 2i withdrawal. Expression levels differing significantly between the two conditions are coloured yellow (P-value <0.05) and red (P-value <0.01). Data are shown graphically on the right.

Table S7. Expression changes caused by Jun depletion and overexpression in ESCs. The 406 genes that were both significantly differentially expressed upon Jun depletion (siJun data) and were expressed in upon Jun over-expression (doxJun data), together with the fold changes ($\log_2$) caused by Jun depletion (siJun) or overexpression (doxJun) compared with their corresponding control data.

Table S8. Primers used for RT-qPCR.
